# Eggshell apex abnormalities caused by two different *Mycoplasma synoviae* genotypes and evaluation of eggshell anomalies by full-field optical coherence tomography

**DOI:** 10.1186/s12917-018-1758-8

**Published:** 2019-01-03

**Authors:** Olimpia Kursa, Anna Pakuła, Grzegorz Tomczyk, Sławomir Paśko, Anna Sawicka

**Affiliations:** 1 Department of Poultry Diseases, National Veterinary Research Institute, Al. Partyzantów 57, 24-100 Pulawy, Poland; 2 Institute of Micromechanics and Photonics, Faculty of Mechatronics, Virtual Reality Techniques Division, Warsaw University of Technology, ul. A. Boboli 8, 02-525 Warsaw, Poland

**Keywords:** *Mycoplasma synoviae*, Apex eggshell abnormalities, Poultry, Full-field optical coherence tomography, EAA

## Abstract

**Background:**

*Mycoplasma synoviae* (MS) is an important poultry pathogen worldwide. This bacterium may cause eggshell changes including an altered shell surface, thinning, and increased translucency in different areas, which leads to a greater incidence of eggshell cracks and breaks. In the present study the association between experimental infection of birds with two field strains of MS from different genotypes and the production of abnormal eggs is described. The analysis of those eggshells using a full-field optical coherence tomography (FF OCT) scanner is also reported.

**Results:**

Eggshell samples were obtained from three experimental groups of chickens: one control and two infected tracheally with field strains of MS which produced abnormal eggs. In both experimental groups infected with MS a reduction of mean daily egg production by 11% was observed compared to the control group, which started at 21 to 42 dpi. Eggshell apex abnormalities increased to 24.5% of eggs and in some cases, soft-shelled eggs were produced. This study provides the first analysis of shells from anomalous eggs carried out using FF OCT, which allows three-dimensional structural imaging of an investigated sample at micrometre scale. FF OCT showed ultrastructural changes in eggshells and a smaller number of pores on the entire surface of the affected shells.

**Conclusions:**

The eggshell pathology and the concomitant egg production losses that result from infections highlight the economic significance of MS in commercial poultry. There are differences in the strains of MS which may induce eggshell apex abnormalities (EAA) and egg production losses. The use of FF OCT, which is a noninvasive measurement method based on analysis of the light backscattered from the measured object, will confer the ability to control the quality of eggshells in flocks infected with MS.

## Background

The pathogenenicity of *Mycoplasma synoviae* (MS) can cause considerable economic loss in the poultry industry. This microorganism is known to cause respiratory distress, synovitis, airsacculitis, and reduced egg production [1]. Infection most frequently occurs sub-clinically and the only effective way to control it is to maintain MS-free breeder flocks. There are differences in the prevalence of eggshell apex abnormalities (EAA) [2] which may be explained by the different tropisms of the infecting MS strains. In recent years, the occurrence of strains of MS which induce EAA and lose egg production was reported in the Netherlands [3], Italy [4]^,^ Germany [5], Australia [6] and Brazil [7]. EAA is characterised by an altered shell surface, thinning, increased translucency, and cracks and breaks in the eggshell. Changes significatory of EAA in egg quality and ultrastructure were also observed in Polish flocks. Antimicrobial treatment of the infection is difficult as MS lacks a cell wall, so the bacterium is refractory to treatment by several antimicrobials including the β-lactams. An MS infection was successfully treated using tetracyclines (doxycycline, oxytetracycline and chlortetracycline) and macrolides (tylvalosin, tylosin and tilmicosin) [2, 8].

The optical coherence tomography (OCT) method has been used for images of the retina and the structures of the human eye [9, 10]. OCT is a non-contact non-destructive method proven to be a reliable and versatile imaging method and has been applied especially in medical sciences but also in many different areas, such as material science [11], archaeology [12], agriculture [13], and veterinary medicine [14]. OCT has also been applied of eggshell thickness measurement [15]. Use of full-field OCT (FF OCT) can not only measure thickness but detect of ultrastructural change in an eggshell and reveal the detailed inner structures of the pores.

This report presents the association between experimentally infected birds challenged with two different field strains of MS and the production of abnormal eggs. This is the first report on the examination of the eggshells with EAA by FF OCT which allows three-dimensional structural imaging of anomalous shells at micrometre scale.

## Methods

### Field study

In the current study MS was isolated from trachea swabs and lung and oviduct tissue from the subject flock by PCR methods and by culture. MS was identified by three techniques: a specific MS PCR [16], LAMP [17] and by sequencing of the *vlhA* gene. Serum antibodies against MS were detected in all samples. Sequencing of the *vlhA* gene from MS-positive samples showed the presence of two different *vlhA* genotypes E and F based on the genotype classification of Dijkman et al. [18]. A strain from genotype F (146-3 J/15PL (MF 737500)) isolated from oviduct tissue was used to infect laying hens. In addition, to compare differences between genotypes, laying hens were also infected with a strain from genotype C (GK1/15PL - MF737474). The strain was isolated from tracheal swabs from a flock where only respiratory distress was observed.

### Experimental infection

Experimental procedures and animal management protocols were carried out in accordance with the detailed unified requirements of the Local Ethics Committee on Animal Experimentation, which also meet the EU standards.

Commercial brown layers at 20 weeks that were serologically negative for MS and *M. gallisepticum* (MG) infection were bought from a poultry farm. They had been vaccinated against Newcastle disease virus (ND), infectious bronchitis virus (IBV) and metapneumovirus. Birds were tested before infection for MG, MS, IBV, ND, avian influenza (AI) and adenoviruses by PCR methods. After 2 weeks’ acclimatization, at the age of 22 weeks the birds were divided into three experimental groups. Each group (*n* = 11) was housed in our experimental infection facility in a different room and maintained with HEPA-filtered air under negative pressure. The room temperature range was 18–20 °C, birds were exposed to 14 h of light per day, and food and drinking water were provided ad libitum*.*

The three experimental groups were inoculated via *trachea*. The first group (*n* = 11) was inoculated with 0.4 ml of broth culture containing 10^4^ colony-forming units (CFU)/ml of the MS strain GK1/15PL isolated from tracheal swabs. The second group (*n* = 11) was inoculated with 0.4 ml of broth culture containing 10^4^ CFU /ml of the MS strain PL146/3-J/15 isolated from oviduct tissue. The third group (control group *n* = 11) was given 0.4 ml of sterile modified Frey Medium.

Tracheal and cloacal swabs were collected from all chickens on days 0, 7, 14, 21, 28, 35, 42, 49, and 56. Blood samples were taken from all birds on the same days for MS serology. Over 8 weeks eggs were collected daily from the control and infected groups and weighed and candled. At the end of the experiment the birds were euthanized, necropsies performed and samples collected as described below. Trachea and *infundibulum*, *magnum* and *uterus* were collected post mortem from both infected groups and randomly selected birds from group III (control group). Tissue taken from trachea and oviduct was cut into small pieces (2 g ± 0.1) and placed in 10 ml of PBS (pH 7.4 ± 0.2) and homogenized. Part of the homogenized tissues were taken for mycoplasma culture and DNA extraction.

Swabs taken from birds were used for general bacteriology and mycoplasma culture. DNA extracted from the MS cultures was used for molecular typing. The shells of unaffected and EAA-affected eggs were examined by FF OCT.

### Serology

Serum samples were examined for MS and MG with a rapid serum plate agglutination test (SPA) using commercial antigens (Soleil Diagnostic (now Ceva Biovac), Beaucouzé, France) [19]. Sera were also analysed for antibodies against MS using an enzyme-linked immunosorbent assay (ELISA) according to the manufacturer’s instructions (IDEXX Laboratories, Westbrook, MA, USA).

### Bacteria and culturing conditions

Swabs from trachea and cloacae and oviduct tissues were inoculated and separately placed in 1 ml modified Frey’s broth [20], for mycoplasma culture. Samples were incubated at 37 °C in a 5% CO_2_ atmosphere for at least 14 days. During this time the cultures were checked daily and when a colour change or turbidity was seen, the broth was inoculated onto Frey’s agar, incubated under the same conditions for a total of 21 days and observed for the presence of typical mycoplasma colonies (“fried eggs”). If no change was seen after 14 days, samples were considered negative.

The polymerase chain reaction (PCR) and loop-mediated isothermal amplification (LAMP) methods were used to confirmed positive mycoplasma cultures.

### Molecular identification

#### DNA extraction

Genomic DNA from MS reference strains and field isolates was extracted using the QIAamp DNA Mini Kit (Qiagen, Hilden, Germany) following the manufacturer’s instructions. The DNA samples were stored at − 70 °C until further analysis.

#### PCR and sequencing

The MS detection primers (MSLF and MSsR) used in this study were previously described by Hammond et al. [16] for identification of the variable lipoprotein and haemagglutinin (*vlhA)* gene. The reaction was carried out in a final volume of 25 μL containing 12.5 μL of Taq PCR Master Mix (EurX, Gdansk, Poland), 10 μM of each MSLF and MSsR primer and 7.5 μL of deionised water. The thermal profile was 94 °C for 2 min, followed by 35 cycles of 94 °C for 30 s, 55 °C for 30 s, 72 °C for 60 s, and a final extension at 72 °C for 7 min. The PCR products were separated in 2% agarose gel stained with ethidium bromide and their length was compared to the 100 bp DNA Ladder Plus GeneRuler (Thermo Scientific, Waltham, MA, USA). PCR products were sequenced using an 8-capillary Genetic Analyzer (3500 Series, Applied Biosystems). Sequence data were assembled and edited with MEGA 7 software.

### Lamp

Loop-mediated isothermal amplification for detection of DNA MS was performed exactly as described by Kursa [17].

### Gross pathology

At post mortem gross oviduct pathology was observed and recorded with particular regard to colour, structure and inflammatory conditions.

### Full-field optical coherence tomography

Analyses of the structure of egg shells with EAA and unaffected eggs were carried out using FF OCT which allows three-dimensional structural imaging of an investigated sample at the micrometre scale. The laboratory FF OCT setup is based on Linnik configuration with a near-infrared light source. The FF OCT applies a matrix detector and lateral scanning of an object is no longer necessary. The main setup parameters were: magnification 54.7 x, field of view diameter 0.3 mm, axial resolution 15 μm, and lateral resolution 2 μm.

### Statistical analysis

Egg weights in grams for the two experimental groups and control group were noted to provide data for statistical analyses. Records were taken for eggs from the second week after inoculation until the end of the experiment. Egg weights for each group in every week of the experiment were compared using the Mann–Whitney test. Comparison between the experimental groups of daily egg production was performed using Pearson’s chi-square test. A *p* value of < 0.05 was considered statistically significant.

## Results

### Experimental study

All samples were negative for MG by serology, culture and PCR and also negative for the main poultry bacterial (ORT, *E. coli*) and viral (IBV, ND and AI) pathogens. No serum antibodies against MS or genetic material were detected in the chicken of origin on the day of infection, and nor were clinical signs of disease observed in any experimental group throughout the study. Antibodies against MS were only detected in the birds inoculated with MS, but no antibodies were detected in the control group. MS was isolated at 14 dpi from tracheal swabs from all infected birds. The isolates remained true to their genotypes. However, mycoplasmas were isolated post mortem at 8 weeks from the oviducts of 6 out of 11 birds infected with strain GK1/15PL and 4 out of 11 birds infected with strain 146-3 J/15PL.

**Fig. 1:**
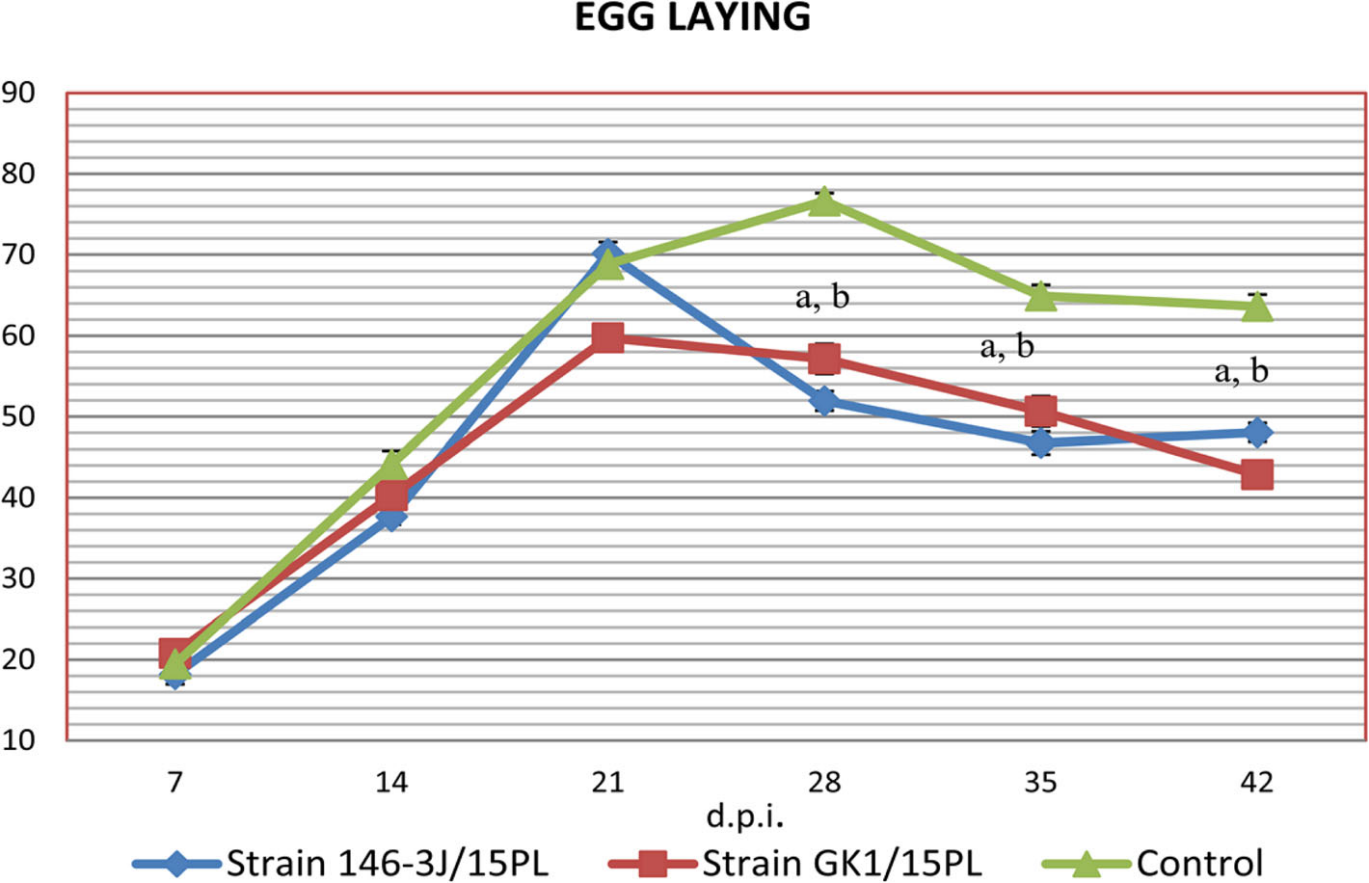
Mean number of eggs produced per bird per week in each group in the experimental study (**a**) *P* < 0.05 between the group infected with strain GK1/15PL and control, (**b**) *P* < 0.05 between the group infected with strain 146-3 J/15PL and the control

**Fig. 2:**
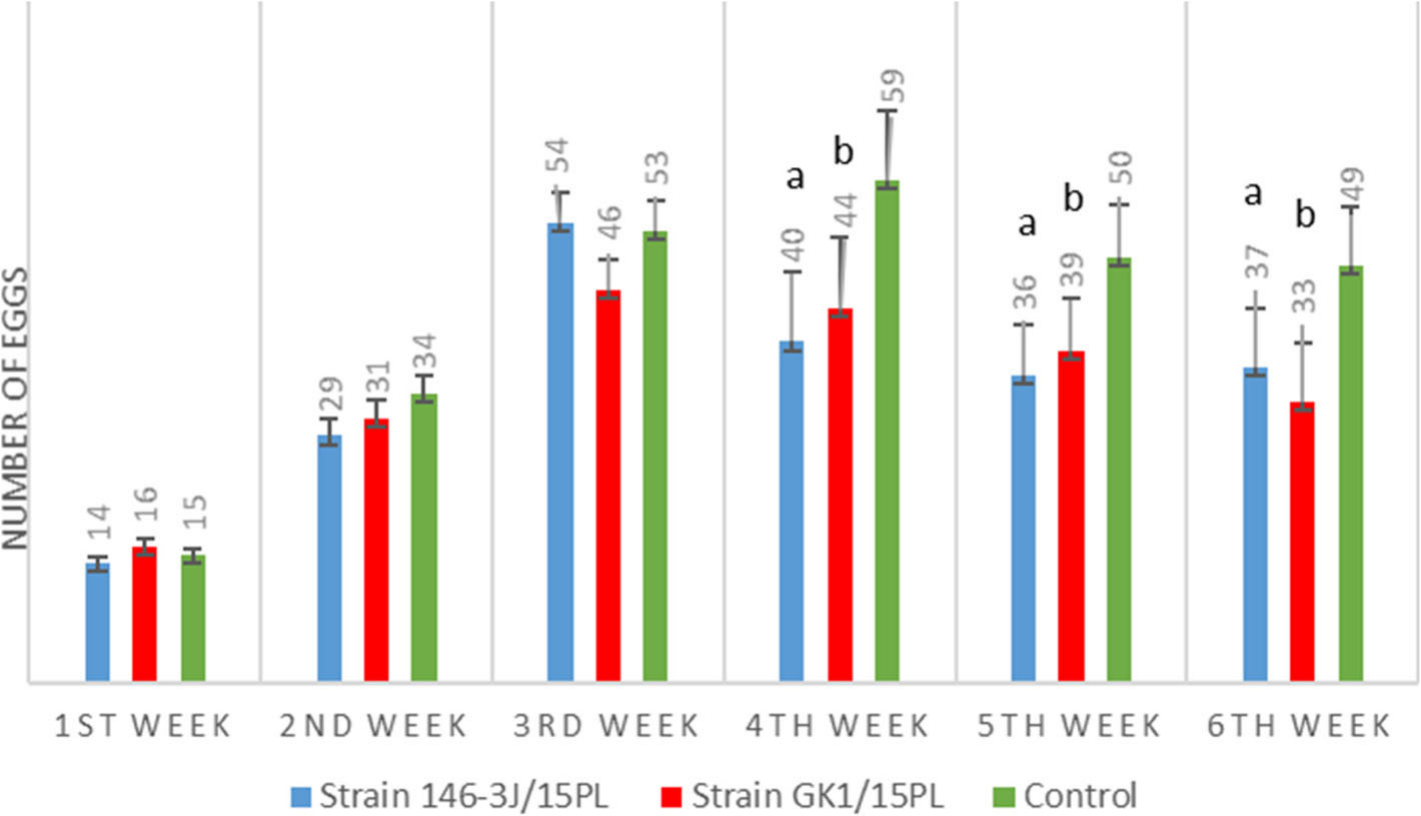
Number of eggs produced per bird per week in each experimental group. (**a**) *P* < 0.05 between the group infected with strain GK1/15PL and the control, (**b**) *P* < 0.05 between the group infected with strain 146-3 J/15PL and the control

Analysis of the daily laying shows that by the end of the experiment the control group (III) had produced a higher mean number of eggs per bird per week than either of the other two groups (I and II) (Fig. 1, Fig. 2). The highest drop of 24% was in group II on day 28 after infection. In group I the highest drop in egg production, of 20.7%, was on day 42 after infection. In both experimental groups infected with MS a decline in mean daily egg production of 11% was observed compared to the control group. The mean daily egg production was significantly lower from 28 dpi and this underproduction lasted up to 46 dpi. A statistically significant difference in laying between the infected groups and control group was demonstrated (*P* < 0.05).

**Fig. 3:**
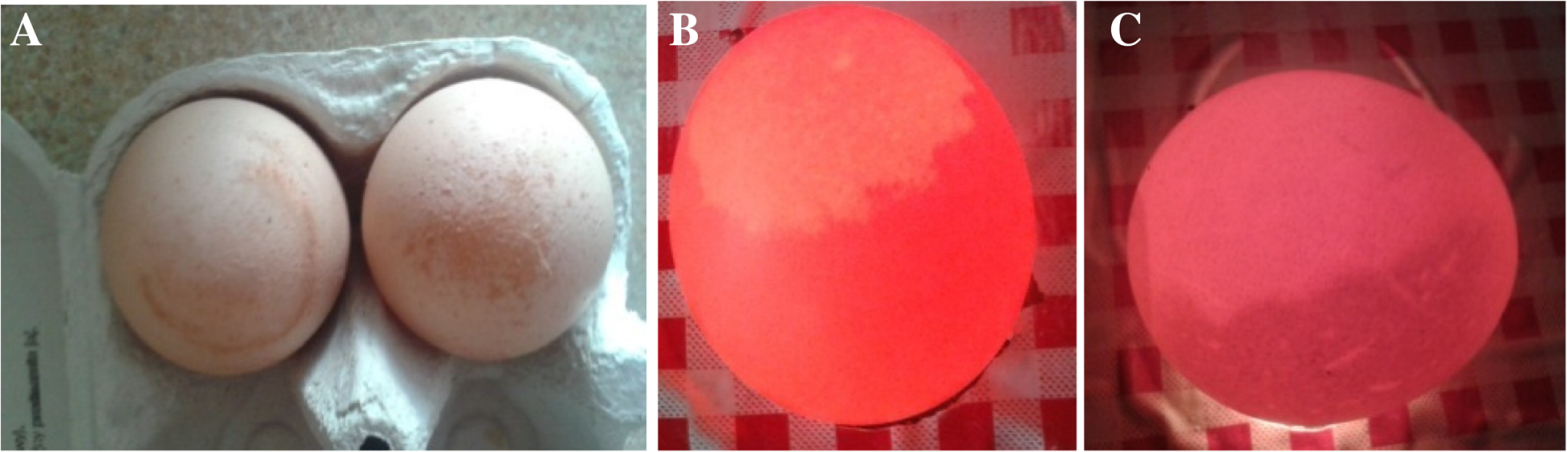
Eggs with EAA characterized by an altered shell surface and increased translucency (**a**). Egg with clear demarcation zone at the apex of the shell at candling (**b**). Egg with increased translucency at candling (**c**)

The production of eggs with EAA started on day 17 in group I and on day 25 in group II after MS inoculation in these groups. Between weeks 1 and 7, 260 unaffected eggs from the control group a lower number of eggs (affected specimens) from each infected group (*n* = 209 and *n* = 210) were tested (Fig. 2). Most eggs with anomalies had the demarcation zone at the apex of the egg (Fig. 3a, b). Characteristic changes were more visible macroscopically and in the candling in group II. In addition to the changes at the apex, the shell thinning and increased translucency in different areas of the eggshell were visible during the candling (Fig. 3c). The frequency of abnormal eggs from affected groups increased to 24.5%. In some cases, soft-shelled eggs were also produced.

Statistical analysis of egg weights showed statistically significant differences (*P* < 0.05) between the group infected with the strain 146-3 J/15PL and the control group in weeks 1, 3, 4, 5 and 6 after infection, while between the group infected with the strain GK1/15PL and the control group, statistically significant differences were found only in the second week after infection. Statistically significant differences between the two infected groups were demonstrated in weeks 2, 4 and 6 after infection (Table 1).

**Table 1:** Egg weight per week in each group in the experimental study

Egg weight (g)			
Week	Strain GK1/15PL	Strain 146-3 J/15PL	Control
1	51,36 ± 1,3	51,24 ± 3,3	53,62 ± 3,9
2	57,23 ± 1,9	54,15 ± 3,2	56,68 ± 1,6
3	55,17 ± 3,3	54,22 ± 3,2	60,02 ± 1,8
4	57,23 ± 4,6	44,31 ± 5,7	59,60 ± 1,5
5	56,29 ± 2,8	54,79 ± 3,0	59,25 ± 1,6
6	54,85 ± 2,7	54,71 ± 1,8	58,16 ± 2,3

At post mortem respiratory tract signs were observed in individual birds in both groups. Macroscopic abnormalities were detected in oviduct in birds producing eggs with EAA. The obtained results require additional research. No gross lesions were observed in the control group.

Strains reisolated from the fallopian tubes of infected hens were assigned in genotypic studies to the F and C genotypes. Strains used for infection did not undergo significant mutations during infection and the similarity between the strain used for the experimental infection of laying hens and those reisolated from the fallopian tubes was 99%.

### FF OCT

**Fig. 4:**
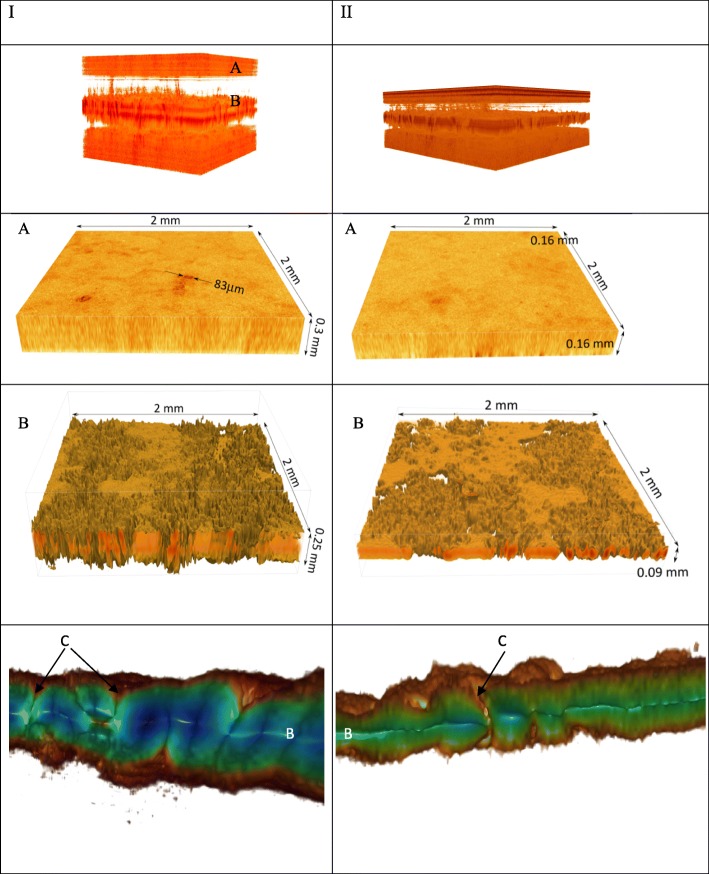
I: FF OCT image of an unaffected eggshell showing the palisade layer (**a**), inner membranes (**b**) and pores (**c**). II: FF OCT image of an abnormal apical eggshell showing the much reduced palisade layer (**a**) and mammillary layer (**b**)

Eggs with EAA were characterised by an altered shell surface, shell thinning and increased translucency (Fig. 3). The eggshell abnormalities were in a region extending approximately 2 cm from the apex of the egg, and in most cases there was a very clear demarcation zone. Eggs with aberrant shells were analysed by FF OCT, which also demonstrated the existence of pathologic changes. The current study describes the first use of the FF OCT method to present changes at the ultrastructure level. The width of the palisade layer was significantly reduced in both groups by about 47% compared to control eggshells (from 0.3 mm to 0.16 mm) (Fig. 4a). The FF OTC analysis of small eggshell samples demonstrated specific changes in the inner membranes of the eggs with EAA. Mammillary knob layers were significantly different in eggshells with changes. A loss of mammillary knob layer detail of about 64% compared to control eggshells (from 0.25 mm to 0.09 mm) was observed for EAA eggs from the two infected groups (Fig. 4b). Also the number of pores in the changed areas was reduced (Fig. 4c).

## Discussion

*M. synoviae* infection in chickens and turkeys causes economic losses to the poultry industry worldwide. The most frequent occurrence of the infections is subclinical upper respiratory infection, which can progress to respiratory disease or to infectious synovitis. Some of the MS strains are able to produce EAA leading to a significant drop in eggshell strength, egg breakage and general decrease in egg production. For the poultry industry it is very important to detect problems related to egg quality, and intactness and high quality of eggshells are paramount [21, 22]. If the eggshell has cracks on its surface it is prone to damage, dehydration, and microbial infection. Such an egg is afflicted with low quality in general. The more frequently these problems occur, the higher embryonic mortality rates are observed [21]. The Dutch [3], Italian [4] and Australian [6] strains of MS were found to be some of the factors causing EAA. There is little information available regarding the effects of Polish strains of MS on eggshell quality.

In the present study we have shown the occurrence of pathological changes after infection of hens with Polish strains of MS belonging to two different genotypes: F and C.

In our experimental infection both strains suppressed the number of eggs produced and produced eggs with EAA (increased incidence of soft-shelled eggs and egg breakage). In both infected groups colonies as well as genetic material were isolated from the oviduct at the end of the experiment (at 8 weeks). In group I, genetic material of MS was found in 54% of samples and in group II in 36% of tested samples. It seems that the strain GK1/15PL caused the highest drop in egg production at a later time (42 dpi), but the eggs with anomalies were already appearing at 17 dpi. However, in group II the mean daily egg production was significantly lower than in the control group at 28 dpi and production of changed eggs started at 25 dpi (Fig. 1, Table 1). This can explain the differences in detected MS DNA in the samples from oviduct tissue. The strain from the first group could induce changes in the oviduct earlier and for longer than strain from the second group which induced a sudden drop in egg laying. However, egg weight was found to be significantly lower in the group infected with strain 146-3 J/15PL as compared to the group infected with strain GK1/15PL. The first eggs with EAA were seen on day 17 post infection. This was earlier than seen in the experimental infection reported by Feberwee et al. [3], when the first EAA manifestation started on day 26 post infection, but was later than seen in the experimental infection described by Catania et al. [4] when eggs with EAA first appeared on the 6th day. This difference could be related to the type of chicken used in the experiment (commercial Bovans Brown layers vs. commercial white layers and white HyLine SPF), or to the genotype of the MS strains. The percentage of abnormal eggs (24.5%) detected in our experimental study was higher than the 10.2% in Brazil and 7.2% in Italy reported previously but similar to the 25% in layers housed on the floor in the Netherlands [2, 3, 23]. Egg production loss in flocks infected with MS over the period of the experiment (10–24.7%) was similar to that seen in flocks in Brazil 10–23% [23].

In our studies we used two strains from two different genotypes (F and C). There were differences between them in the time of drop in egg production and egg weight. Both infected groups produced eggs with a very clear demarcation zone in the region extending approximately 2 cm from the apex of the egg (Fig. 4a, b). However, in group II there were more eggs with ultrastructural changes not only in the apex but also in different areas of the shell (Fig. 4c). The damaging effects of the MS on the hen reproductive organs were evaluated with gross autopsy during the 8-week observation after infection. The mechanism by which MS affects eggshell calcification is not clear although its elucidation has already been attempted by several authors [2, 3, 6, 23, 24]. Many factors can affect the quality of the shell. Studies conducted by Catania [4] reported that not all strains are capable of causing EAA, Feberwee et al. [3] reported synergism between MS and IBV and lower occurrence of MS-induced EAA following administration of the live vaccine MS-H, but the study findings of Gole et al. [6] reported a high translucency score in the vaccinated group.

## Conclusions

To our knowledge, this is the first study demonstrating the use of FF OCT to present the pathology of the eggshell with EAA. Eggs from hens infected with MS showed their shells to be severely diminished, which was in agreement with the translucency detectable macroscopically, particularly at candling. Study of eggshells with FF OCT provided an explanation for the diminished eggshell having greater translucency. Ultrastructural changes were found in the apical eggshell and in other parts of it. In addition, the studies showed a smaller number of pores on the entire surface of the shell, which can cause very high embryonic mortality.

## Abbreviations

EAA: Eggshell apex abnormalities
FF OCT: Full-field optical coherence tomography
HEPA: High efficiency particulate air filter
MS: *Mycoplasma synoviae*,
OCT: Optical coherence tomography
SPF: Specific-pathogen-free
*vlhA*: Variable lipoprotein hemagglutinin A
